# Segmentation of overweight Americans and opportunities for social marketing

**DOI:** 10.1186/1479-5868-6-13

**Published:** 2009-03-08

**Authors:** Jane Kolodinsky, Travis Reynolds

**Affiliations:** 1 Department of Community Development and Applied Economics, University of Vermont, Burlington, VT, USA; 2 Evans School of Public Affairs, University of Washington, Seattle, WA, USA

## Abstract

**Background:**

The food industry uses market segmentation to target products toward specific groups of consumers with similar attitudinal, demographic, or lifestyle characteristics. Our aims were to identify distinguishable segments within the US overweight population to be targeted with messages and media aimed at moving Americans toward more healthy weights.

**Methods:**

Cluster analysis was used to identify segments of consumers based on both food and lifestyle behaviors related to unhealthy weights. Drawing from Social Learning Theory, the Health Belief Model, and existing market segmentation literature, the study identified five distinct, recognizable market segments based on knowledge and behavioral and environmental factors. Implications for social marketing campaigns designed to move Americans toward more healthy weights were explored.

**Results:**

The five clusters identified were: *Highest Risk *(19%); *At Risk *(22%); *Right Behavior/Wrong Results *(33%); *Getting Best Results *(13%); and *Doing OK *(12%). Ninety-nine percent of those in the *Highest Risk *cluster were overweight; members watched the most television and exercised the least. Fifty-five percent of those in the *At Risk *cluster were overweight; members logged the most computer time and almost half rarely or never read food labels. Sixty-six percent of those in the *Right Behavior/Wrong Results *cluster were overweight; however, 95% of them were familiar with the food pyramid. Members reported eating a low percentage of fast food meals (8%) compared to other groups but a higher percentage of other restaurant meals (15%). Less than six percent of those in the *Getting Best Results *cluster were overweight; every member read food labels and 75% of members' meals were "made from scratch." Eighteen percent of those in the *Doing OK *cluster were overweight; members watched the least television and reported eating 78% of their meals "made from scratch."

**Conclusion:**

This study demonstrated that five distinct market segments can be identified for social marketing efforts aimed at addressing the obesity epidemic. Through the identification of these five segments, social marketing campaigns can utilize selected channels and messages that communicate the most relevant and important information. The results of this study offer insight into how segmentation strategies and social marketing messages may improve public health.

## Background

It is no longer news that unhealthy eating behaviors and sedentary lifestyles have contributed to the current obesity epidemic in the United States. However, the 66 percent of Americans who are overweight do not form a homogeneous group – attitudes, demographic characteristics and lifestyle choices vary greatly within this subset of the US population. Segmentation theory tells us that a "one size fits all" approach to marketing social change may not meet the needs of all people. Further, marketing research has revealed the importance and effectiveness of tailoring messages and incentives to meet the needs of different population segments. "Social marketing" is defined as "a social change campaign organized by a group which intends to persuade others to accept, modify or abandon certain ideas, attitudes, practices or behavior" [1]. A social marketing campaign using market segmentation may be one effective tool for helping move more Americans toward healthier weights [2].

The food industry has used market segmentation of consumers for decades. As early as 1950, Haire segmented consumers based on personality characteristics in order to increase the sales of instant coffee [3]. Today, more than half a century later, segmentation is still being used to market twenty-first century foods to consumers [4,5]. Even the dairy industry has engaged in segmentation in an effort to increase sales of dairy products based on research that links the consumption of dairy foods to weight loss [6]. Segmentation has enabled the industry to target its products toward specific groups of consumers with similar attitudinal, demographic, or lifestyle characteristics.

The success of segmentation strategies for food marketing suggests that such techniques may hold promise for identifying ways to change consumer behavior regarding unhealthy food and lifestyles [7]. Psycho-behavioral segmentation – or segmenting on the basis of what people are doing (i.e., the behavior), and why (i.e, the social and psychological antecedents to the behavior) – has already been employed for health promotion research focusing on alcoholism [8] and overall health [9,10]. In some instances segmentation has even been explicitly tied to social marketing efforts: "5 a day for better health", for example, is a social marketing campaign that encourages more positive nutrition behaviors among American consumers [8]. The "5 a day" campaign helped increase the percentage of Americans consuming five or more servings of fruits and vegetables per day from 23 percent in 1991 to 35 percent in 2003 [11]. To achieve this, the campaign recognized and made use of the existence of market segments, both demographic and psychosocial [12]. Recent reports by the US Department of Health and Human Services and the National Institutes of Health further highlighted the need to identify specific population segments for targeted interventions in the fight against obesity, including efforts to assess how obesity-related knowledge, behavior, and environments may affect consumer behavior [13,14].

Segmentation is used by marketers because it works. Not every individual is a potential consumer of a given product, idea or service, so tailoring messages to specific groups can be more effective than broadcasting to everyone. Consumers are segmented based on geographic location, demographic characteristics, and product use. Contemporary marketers now also employ lifestyle-based and product benefit approaches [6,8-10,15].

While the techniques of market segmentation have long been used in the field of for-profit marketing, they have only recently been used as tools to help meet the goals of social marketing campaigns. One impediment to widespread adoption of market segmentation strategies is that social marketing segmentation studies related to healthy food behaviors have not shown consistent results across demographic, behavioral and lifestyle variables [1,11]. A wealth of empirical research has linked demographic characteristics, dietary behaviors, media habits, and psychological variables to overweight status – for a thorough review of this literature, see Jeffery & Utter [16], Ball *et al*. [17], or Trudeau *et al*. [12]. However, though some studies have linked factors such as socio-economic status, gender, and dietary patterns to overweight [18-21], others have not [22,23]. Ultimately, there remains a certain degree of uncertainty in the scientific community about how the energy imbalance leading to increased body weight among Americans is occurring [16]. This suggests that while previous research can provide some guidance as to the types of variables that should be included in a segmentation study of overweight in the US, health advocates cannot simply interpolate past results to shape social marketing campaigns for changing health behaviors.

Social Learning Theory [24], the Health Belief Model [25], and their offshoots have been proposed as theoretical frameworks suited to the application of market segmentation in studies of consumer health behavior change [7,26]. Such models typically include both personal and environmental variables [26]. By way of example, Miles *et al. *[27] examined a mass-media health campaign in the United Kingdom based on both Social Learning Theory and the Health Belief Model. Through the analysis of the results of this campaign a clear indication of market segments emerged including demographic segments characterized by socio-economic status, age, gender, and overweight [27]. Following the campaign, men reported larger lifestyle changes than women. Those in lower income categories were less likely to be aware of the campaign. Such information can be invaluable for reformulating future campaigns to ensure that they are more effective.

In the US, Loughrey *et al*. used segmentation techniques in the form of audience-profiling to promote the 2000 Dietary Guidelines for Americans [26]. Using national market data, the researchers delineated three market segments based on the Healthy Eating Index: Better Eaters, Fair Eaters and Poor Eaters. In addition to demographic characteristics, beliefs, values, and both food and media habits were used in the segmentation process. Demographic variables explained little regarding differences across segments, and there were no differences between the media habits of the groups. However, dietary choices and attitudes did prove significant. Better Eaters were more likely to take action to eat a healthy diet and were better able to anticipate outcomes of their behaviors. Poor Eaters were less likely to worry about the nutritional content of foods. Fair Eaters fell in the middle of these two extremes. Based on these segments, three distinct message-development strategies were undertaken. It was determined that Better Eater messages might best focus on simple, positive messages to help maintain healthy eating behaviors. Fair Eaters were in need of messages that would precipitate action to change eating behaviors. A highly targeted approach was recommended for Poor Eaters; one which both captured attention and established 'cultural relevance' [26].

Qualitative focus group research is another approach that has been employed to gain insights into how to communicate appropriate health-related messages to consumers, especially messages based on the 2005 Dietary Guidelines for Americans [28]. Focus groups including segments based on gender, overweight, and age have shown that messages to the public must be inclusive, trustworthy, and not "too markety" [28]. These results point to the need to further segment the population beyond demographic characteristics. Although messages must be inclusive, they must also be applicable to different sub-segments of the population, each of which may have differing lifestyles or may draw upon different sources of information.

Today segmentation is emerging in more modern incarnations that move beyond social marketing. The Internet has enabled marketers to refine segmentation to the level of microsegments, made possible in part because web users' behavior can be tracked more readily and unobtrusively than that of traditional consumers [29,30]. The Internet also provides the medium to act on these narrow segments through unique offerings that appeal to these narrow segments [29]. Similarly, political campaigns have recently attributed some of their successes to data mining technology used to identify and segment like-minded individuals and craft uniquely appealing messages to targeted segments about their candidate, dubbed "microtargeting" [31,32]. Some argue that political marketing [33] and accompanying meaningful segmentation represents an avenue that must be traversed in any successful campaign [32]. Though the implications of these new and expanding marketing channels have yet to be explored in a social marketing context, "microsegmentation" and "microtargeting" may provide opportunities for more effective health behavior promotion in modern societies.

An effective fight against obesity must coordinate public and private campaigns against unhealthy food choices. This requires developing and delivering clear, coherent health messages and developing targeted programs on specific segments of the population [13]. Ultimately, there is emerging evidence that the development of targeted messages based on segmentation of the population holds promise for a social marketing campaign seeking to promote healthier dietary and lifestyle choices [1,3]. Empirical research in this area remains in its infancy, but available research does provide insights into theoretical foundations and empirical measures appropriate for use in social marketing segmentation studies.

This study uses cluster analysis to identify different segments of US consumers based on food choices, activity, food knowledge, overweight, and other environmental variables. The goal of the analysis is to identify distinguishable segments with the U.S. overweight population that can be reached with appropriate messages and media channels aimed at moving them toward more healthy weights.

## Methods

### Study population

Data used in this study are from a national poll, funded by a United States Department of Agriculture Grant. The questionnaire was administered over a two-week period by trained staff using a computer-aided telephone interviewing system (CATI). Each interview took approximately 15 minutes to complete, and up to five callbacks were made to households. A geographically stratified random sampling technique was used. Seven regions of the United States were identified: Northeast; Mid-Atlantic; South; Great Lakes; North Central; South Central; and Pacific. Land-line telephone numbers for each of the seven regions were purchased from infoUSA^®^. InfoUSA^® ^provides consumer contact lists. They catalog telephone directories to compile extensive, comprehensive databases of consumer information that are updated monthly and sold to market researchers. A random number generator was used within each region to generate the final sample size of 581. Only adults over the age of 18 were eligible to complete the survey; approval for the study was obtained from the Institutional Review Board at the University. Power calculations reveal that the sample size resulted in a 95% probability of detecting a 5% difference in the dependent variable with 95% power. Demographic characteristics of the sample are provided in Table 1.

**Table 1:** Description of the Sample

Behavioral Variables	Variable Description	Summary Statistic (n = 581)
*Knowledge*		
Know Pyramid	Know food pyramid = 1	0.83^a^
Read Labels	Most of the time, sometimes, rarely, never	Most of the time^b^
		
*Type of Labels Read*		
Calories	Read calories label = 1	0.48^a^
Carbohydrates	Read carbohydrates label = 1	0.23^a^
Fat	Read fat label = 1	0.48^a^
Protein	Read protein label = 1	0.08^a^
Salt	Read salt label = 1	0.18^a^
Serving size	Read serving size = 1	0.05^a^
Sugar	Read sugar content = 1	0.22^a^
		
*Food Behavior*		
Overweight	Overweight = 1	0.53^a^
Calories right	More, less, about right amount	Less than needed^b^
Eat well	Always, sometimes, never choose a healthy diet	Sometimes choose a healthy diet^b^
Eat weight	Eating to lose weight = 1	0.55^a^
Fast food meals	Percent fast food meals	12.2 (18.0)^c^
Restaurant meals	Percent restaurant meals	12.8 (15.6)^c^
Prepared meals	Percent prepared food meals	6.9 (13.0)^c^
Scratch meals	Percent meals from scratch	67.0 (27.4)^c^
		
*Risk Factors*		
Smoker	Smoker = 1	0.17^a^
Illness	Illness limits activity = 1	0.24^a^
Insurance	Covered by insurance = 1	0.89^a^
		
*Activity*		
TV	Minutes of TV	123.5 (101.6)^c^
Computer	Minutes of home computer	52.9 (74.6)^c^
Exercise	Exercise 30 minutes/day, 5 times/wk = 1	0.66^a^
Exercise weight	Using exercise to lose weight = 1	0.49^a^
		
*Demographic Variables*		
*gender*	Gender (1 = male)	0.45^a^
*education*		
	Less than HS = 1	0.02^a^
	HS grad = 1	0.08^a^
	Some college = 1	0.53^a^
	Bachelors or more = 1	0.37^a^
*income*		
	Less than $20,000 = 1	0.13^a^
	$20,001 to $35,000 = 1	0.18^a^
	$35,001 to $50,000 = 1	0.17^a^
	$50,001 to $65,000 = 1	0.15^a^
	$65,001 or greater = 1	0.38^a^
*children*	Has children = 1	0.42^a^
*Employment*		
employed	Employed = 1	0.55^a^
unemp	Unemployed = 1	0.04^a^
retired	Retired = 1	0.18^a^
*age*	Age in years	45.27 (17.8)^c^
*Region*		
neast	Northeast = 1	0.28^a^
south	South = 1	0.27^a^
midamer	Middle America = 1	0.26^a^
west	West = 1	0.21^a^
*Urban characteristic*		
rural	Rural = 1	0.32^a^
suburb	Suburban = 1	0.75^a^
urban	Urban = 1	0.25^a^

n = 581

^a ^dummy variable 0/1

^b ^median reported;

^c ^continuous variable, standard deviation in ()

### Measures

The survey instrument was designed to collect information about both personal and environmental characteristics. While no segmentation study can completely measure all the respondent characteristics related to obesity or healthy lifestyle behaviors, our questionnaire attempted to collect data on a wide array of characteristics and behaviors drawn from Social Learning Theory and the Health Belief Model, or found to be significant in other studies [12,16,26]. Socio-demographic characteristics included *gender, education, income, children, employment status, age, geographic region *and *urban/rural residence*.

Body mass information was collected in such a way as to minimize under-reporting of overweight among survey respondents: respondents were first asked their height, and then the CATI system automatically branched to a single question on weight that corresponded to the CDC standard calculations of body mass index (BMI) by height and weight [34]. For example, if a respondent was a female with a height of 5 feet 6 inches (66 inches), the CATI system branched to a question asking, "Do you weigh less than 155 pounds?" If the respondent indicated "yes," they were classified as not overweight. If they answered "no," they were classified as overweight based on the CDC body mass tables [34].

The instrument also collected data on leisure time physical activity, including whether respondents obtained the recommended 30 minutes of exercise five days per week (*exercise*). Sedentary behavior was measured through questions about computer use (*computer*) and television watching (*TV*). Health related variables included whether respondents had a chronic illness limiting their activity (*illness limits activity*), whether they were covered by a health insurance plan (*insurance*) and whether they smoked (*smoker*). Question wording was based on the CDC's Behavioral Risk Factor Surveillance System Questionnaire [35]. A proxy for food knowledge was created based on respondents' food information searching behavior. Respondents were asked how often they read food labels (*read labels*) and what type of information they looked for on a label. This information was obtained through a series of yes/no questions about respondents' label-reading behavior related to calories (*calories*), carbohydrates (*carbohydrates*), fat (*fat*) protein (*protein*), salt (*salt*) serving size (*serving size*), and sugar (*sugar*). Respondents were also asked whether they had heard of the food pyramid (*know pyramid*). MyPyramid was not yet introduced at the time of the survey [36].

Motivations about diet and exercise were collected based on the questions "Do you get at least 30 minutes of exercise five days a week?" (*exercise*), "Are you exercising to lose weight?" (*exercising weight*) and "Are you eating to lose weight?" (*eating weight*). Respondents were also asked whether they were eating an appropriate number of calories per day (*calories right*) and whether they "most often choose a healthy diet," "sometimes choose a healthy diet," or "eat pretty much what they want" (*eat well*). Information on other food behaviors included the number of meals eaten at home (both prepared from scratch (*scratch meals*) and purchased prepared (*prepared meals*)), fast food meals (*fast food meals*), and number of other restaurant meals (*other restaurant meals*) in the last week. For each individual the total number of weekly meals consumed was calculated and percentage of meals from each source was derived to standardize across respondents. Descriptive statistics are provided in Table 1.

### Statistical analyses

Two Step Cluster Analysis using Schwart's Baysian Criteria in the Statistical Package for Social Sciences (SPSS 12.0.1) was used to identify clusters of respondents. This technique is an exploratory method used for both continuous and categorical data. Demographic variables were omitted from the cluster analysis, so that cluster membership was driven by respondent behaviors rather than demographic characteristics. Once the clusters were identified, bi-variate tests of association (ANOVA and Chi-square depending on level of measurement) were used to determine whether cluster membership was associated with demographic characteristics.

## Results

### Cluster Analysis

Five clusters were identified based on overweight status, information search, activity level, health indicators, and food behaviors. Three of the five clusters were characterized as overweight, comprising almost three-quarters of the sample. The remaining two clusters (the remaining 25 percent of the sample) were characterized as not overweight. Table 2 describes the characteristics of each of the clusters. The clusters can be summarized as follows:

**Table 2:** Behavioral Characteristics of Clusters

VARIABLE/CLUSTER	CLUSTER 1: "Highest Risk" (n = 110)	CLUSTER 2: "At Risk" (n = 128)	CLUSTER 3: "Right Behavior/Wrong Results" (n = 192)	CLUSTER 4: "Getting Best Results" (n = 76)	CLUSTER 5: "Doing OK" (n = 75)
***Knowledge***					
Know Pyramid (%)	90.5	69.3	94.6	92.7	86.3
Read labels (%)					
Always	28.6	1.5	44.4	22.2	26.0
Most of the time	29.8	8.8	43.2	38.9	31.6
Sometimes	38.1	43.4	7.1	38.9	27.4
Rarely/Never	3.5	46.3	5.3	0.0	12.0
Type of Label					
Calories (% that read)	43.4	39.0	66.7	58.2	13.8
Carbohydrates (% that read)	15.7	2.9	40.2	20.0	22.4
Fat (% that read)	60.2	14.7	60.9	81.8	53.4
Protein (% that read)	2.4	1.5	17.2	1.8	6.9
Salt (% that read)	20.2	2.2	24.9	10.9	31.0
Serving Size (% that read)	2.4	0.0	8.3	1.8	3.4
Sugar (% that read)	21.4	6.6	30.8	20.0	41.4
					
***Food Behavior***					
Overweight (%)	98.8	53.7	62.1	5.5	17.5
Calories					
% More than needed	9.6	19.7	21.9	3.6	22.8
% About needed amount	66.3	36.5	35.5	7.3	3.5
% Less than needed	14.6	33.6	42.6	87.3	61.4
Eat Healthy					
% Most Often Eat Healthy	22.9	10.3	56.2	61.8	74.6
% Sometimes Eat Healthy	50.6	30.1	33.7	38.2	16.9
% Eat What I Want	26.5	59.6	10.1	0	8.5
Eating weight					
(% eating to lose weight)	66.3	25.5	87.0	88.9	8.6
Fast Food Meals					
(% of meals per week; [#]^a^)	11.7 [2.5 per week]	22.1 [4 per week]	8.4 [2 per week]	8.4 [2 per week]	6.0 [1 per week]
Other Restaurant Meals					
(% of meal per week; [#]^a^)	14.2 [3 per week]	11.5 [2.5 per week]	15.3 [3 per week]	11.4 [2 per week]	10.8 [2 per week]
Prepared Food Meals					
(% of meals per week; [#]^a^)	8.5 [1.5 per week]	9.8 [2 per week]	5.5 [1 per week]	5.3 [1 per week]	5.3 [1 per week]
Scratch Meal					
(% of meals per week; [#]^a^)	65.5 [14 per week]	56.5 [12 per week]	70.8 [15 per week]	74.9 [16 per week]	78.0 [16 per week]
***Risk Factors***					
Smoker (%)	10.7	27.0	10.1	1.9	15.5
Illness					
(% Illness limits activity)	39.8	25.0	18.5	10.9	31.0
Insurance					
(% Covered by insurance)	92.9	82.5	94.6	96.4	86.2
***Activity***					
Sedentary Behavior:					
Television (minutes/day)	157	130	107	131	96
Computer Use (minutes/day)	8	45	47	62	58
Exercise					
(% Exercise regularly)	20.5	75.0	75.0	83.6	73.7
Exercising weight					
(% Exercise to lose weight)	28.9	29.9	88.2	72.2	12.1

^a ^The number of meals per week from each category (in []) is an approximation based on the average total weekly meals consumed by cluster members. All have been rounded to the nearest 0.5 meals.

Overall n = 581

#### Highest Risk

Nineteen percent of the sample fell into this category. Ninety-nine percent of this cluster is overweight, 90% read food labels at least some of the time, and a majority (60.2%) look at fat content. Two thirds believe that they eat "about the right number of calories per day," and more than half "sometimes choose a healthy diet." Eighty percent get little or no exercise, and three quarters are not using exercise to lose weight. Over 40% reported that "chronic illness limits activity." This cluster watches more television than others (more than 2 1/2 hours per day), and eats almost 35% of meals in the form of fast food, other restaurant food, and prepared foods.

#### At risk

Twenty-two percent of the sample fell into this category. Fifty-five percent of the cluster is overweight. Almost half rarely or never read food labels and 30% are unfamiliar with the food pyramid. Sixty percent indicate they "eat what they want." Three fourths report getting at least 30 minutes of exercise five days a week, but 70% are not using exercise to lose weight. Compared to other clusters, more members of this segment are smokers (27%) and do not have health insurance (nearly 20%). This cluster reported eating 22% of their meals as fast food and eating 53% of their meals at restaurants or as prepared foods.

#### Right Behavior/Wrong Results

Thirty-three percent of the sample fell into this category. Almost two-thirds of the cluster is overweight. Eighty percent report they "always read food labels," and most of the group looks for a wide variety of label information. Ninety-five percent of respondents in this group know what the food pyramid is. Eighty-five percent report they eat "less or about the right number of calories," and more than half report "choosing a healthy diet." The majority of this cluster is dieting and exercising to lose weight. This group eats a low percentage of fast food meals (8%), but a higher percentage of other restaurant meals (15%) compared to other clusters.

#### Getting Best Results

Thirteen percent of the sample fell into this category. Less than six percent of cluster members are overweight. Everyone in this group reported reading labels, and 93% know what the food pyramid is. Almost 90% of this cluster reported eating fewer calories than they need and not one reported that they "eat what they want". Ninety percent eat to lose weight and 80% exercise five times per week. This cluster watches less television than the others, and members report eating 75% of their meals "made from scratch."

#### Doing OK

Twelve percent of the sample fell into this category. Within this cluster, 18% of respondents are overweight. Eighty percent read food labels always or most of the time, but most focus on fat and salt content over other information. Seventy-five percent reported choosing a healthy diet and two-thirds reported eating fewer calories than they need. Members of this cluster are not dieting, and 90% are not exercising to lose weight. Almost one-third stated that chronic illness limits their activity. Fifteen percent of this cluster smokes. Members of this segment use home computers about one hour per day, but watch the least television of all groups (96 minutes per day). They eat 78% of their meals "made from scratch." This group also eats the least fast food (six percent of meals) compared to the other clusters.

### Bi-variate Analysis

Bi-variate analysis was used to test whether any demographic characteristics are related to cluster membership. Table 3 presents the results of ANOVA and Chi-square analyses. Gender, income quintiles, age, education and region of residence were found to be significantly related to cluster membership.

**Table 3:** Bivariate Analysis of Cluster Membership by Demographic Characteristics

VARIABLE/ CLUSTER	CLUSTER 1: "Highest Risk"	CLUSTER 2: "At Risk"	CLUSTER 3: "Right Behavior/ Wrong Results"	CLUSTER 4: "Getting Best Results"	CLUSTER 5: "Doing OK"	P value^a^
***Gender ***(% female)	53.0	45.6	59.8	72.2	62.1	0.008 **
***Education ***(%)						0.000 ***
Less than high school	0.0	3.7	0.0	0.0	0.0	
High school grad	2.4	11.0	3.6	11.1	5.3	
Some college	54.8	58.1	56.5	31.5	49.1	
Bachelors or more	42.9	27.2	39.9	57.4	45.6	
***Income ***(%)						0.000 ***
Less than $20,000	17.3	20.2	5.6	7.1	14.5	
$20,001 to $35,000	10.7	21.1	16.0	11.9	32.7	
$35,001 to $50,000	16.0	18.4	9.0	14.3	16.4	
$50,001 to $65,000	16.0	18.4	17.4	9.5	9.1	
$65,001 or greater	40.0	21.9	52.1	57.1	27.3	
***Children ***(% has children)	37.3	45.3	48.2	45.5	32.8	0.216
***Employment ***(%)						
Employed	53.0	46.0	43.5	58.2	79.3	0.000 ***
Unemployed	2.4	3.7	0.6	3.6	3.4	0.417
Retired	27.7	13.2	13.6	23.6	19.0	0.027 *
***Age range ***(%)						0.000 ***
16–24	0.0	19.0	9.5	12.7	10.2	
25–34	15.7	23.4	17.9	16.4	30.5	
35–44	19.3	23.4	27.4	16.4	10.2	
45–54	26.5	12.4	21.4	16.4	18.6	
55–64	12.0	8.8	11.9	12.7	11.9	
65 or more	26.5	13.1	11.9	25.5	18.6	
***Region ***(%)						0.001 ***
Northern	27.7	28.7	30.2	31.5	19	
South	19.3	27.2	23.1	29.6	46.6	
Middle America	16.9	27.9	30.2	20.4	22.4	
West	36.1	16.2	16.6	18.5	12.1	
***Urban characteristic ***(%)						0.163
Rural	30.1	26.3	33.9	23.6	38.6	
Suburban	43.4	41.4	45.8	54.5	45.6	
Urban	26.5	32.3	20.2	21.8	15.8	

^a ^Chi^2 ^statistic; Overall n = 581

* P < 0.05

** P < 0.01

*** P < 0.001

Almost three quarters of the *Getting Best Results *cluster are women, as are two-thirds of the *Doing OK *cluster. Over half of the *Right Behavior/Wrong Results *cluster are men. With regard to income, half of the *Highest Risk *cluster had household incomes of above $50,000. Of those, most were in the highest income group. In contrast, over 40% of the *At Risk *group had incomes under $35,000 per year, and over 60% made less than $50,000. Of the *Right Behavior/Wrong Results *cluster, about 70% had incomes over $50,000. The *Getting Best Results *cluster is also a high income group, with almost 60 percent of this cluster reporting incomes over $65,000 per year. The income levels of the *Doing OK *cluster were more evenly distributed: almost one third reported incomes of between $20,000 and 35,000 per year, while another 30% reported incomes of over $65,000 per year. Age was also significantly related to some clusters' membership: almost half of the *Highest Risk *and *Right Behavior/Wrong Results *clusters are between the ages of 35 and 55. Almost half of *At Risk *cluster members are between the ages of 25 and 45. The *Getting Best Results *and *Doing OK *clusters have a more even age distribution. Finally, more than a third of the *Highest Risk *cluster lives in the West.

## Discussion

This study demonstrates that five distinct, recognizable market segments can be identified for social marketing efforts aimed at addressing the obesity epidemic. Through the identification of these segments, social marketing campaigns can target individuals in order to more successfully communicate the most relevant and important information. The demographic, behavioral, and knowledge characteristics of the five segments identified here imply some strategies for communicating appropriate information to each of the groups.

The findings suggest that *Highest Risk *consumers may benefit from public service messages that remind them of "mindful eating," food choice, and activity as a part of a healthy lifestyle, as it appears they do not pay much attention to the foods they eat, and they exercise the least. Understanding that physical body image, behavioral self-image and self-efficacy can create perceived barriers to exercise [37,38], exercise promotion and support for this group may be appropriate to help address the combined challenges of limited exercise experience and current overweight status. This group could also potentially benefit from basic information about calories and nutritional label interpretation: although the *Highest Risk *group reports reading all label types more than members of the *At Risk *cluster, it is possible that they are still misinterpreting (or not using) this information when making purchases and eating decisions. Finally, the data suggest that the *Highest Risk *cluster watches the most television of any cluster, suggesting these information channels may be the best way to communicate messages tailored to this specific group. Because we do not know more about specific television programming consumed by the Highest Risk group, prime-time television programming would be a sensible start to communicate food choice, exercise, and food label-use education messages.

Meanwhile, it appears that the *At Risk *group would benefit most from messages that improve their overall dietary selections. Members of this segment eat almost half their diet away-from-home, and the majority reports they "eat what they want". Social marketing efforts must seek to successfully communicate the relationships between nutritional intake, health and impending risks to this group. *At Risk *group members must also be informed where to find nutrition information about the foods they are consuming in restaurants. This may be one important factor resulting in higher energy intakes within this segment. The data further show that the *At Risk *cluster logs more computer time than other clusters; tracing computer "cookies" might thus help in micro-targeting of the *At Risk *group. Combined, internet and television channels together would have the greatest reach for the almost 41% of the sample that fell into the *At Risk *and *Highest Risk *segments.

The *Right Behavior/Wrong Results *group reported a basic understanding of food knowledge and appears to be reading food labels more than any other segment. However, over 60% of this segment's members are overweight. Clear, direct messages containing steps that individuals can take to use available nutrition information effectively may work for this group. Given the frequency with which members of this group dine out at restaurants, nutritional messages in restaurant venues might be one way to reach this group in particular. Another possibility, however, is that the *Right Behavior/Wrong Results *group maintains incorrect perceptions about dietary choices and exercise: members of this group may thus be reporting they are making efforts when in fact their definitions of eating healthy foods and engaging in physical activity do not agree with conventional norms. Communicating specific goals and benchmarks to members of this cluster – along the lines of the "5 a day for better health" social marketing campaign in the US [8] – might help overcome such misperceptions.

Finally, the *Getting Best Results *and *Doing OK *groups eat the majority of their meals "made from scratch." These groups are of a healthy weight and perhaps just need to have their healthy food behaviors reinforced. However, the *Doing OK *group also contains the second highest percentage of smokers and has a high percentage of respondents that said chronic illness limited their activity. Members of this group may benefit from broader health messages, as well as specific information on less-intense exercise techniques for individuals who wish to continue an exercise regime in spite of illness. With this segment it is particularly important to acknowledge the risks associated with many non-healthy behaviors such as smoking, which can reduce appetite and thus induce weight loss (resulting in an apparently healthy weight, when the individual in question may not be 'healthy' at all). Further messages to discourage the use of tobacco and other substances to promote weight loss may be of value for this cluster.

The main strength of this study is the isolation of key message and media channel segmentation strategies that may help address the current obesity epidemic. The analysis also raises some important areas for future research and may inform future public policy efforts aimed at combating obesity in the US. Nevertheless, as with previous studies, although some demographic differences are significant between the five groups identified here, there are overweight people in all demographic groups [12], suggesting that there is no "single message" or "single target" for social marketing efforts. Ultimately, while we can make suggestions on the types of messages that might prove to be useful for each segment, more in-depth research is needed within any targeted segment in order to determine specific message strategies that are likely to be most effective. It must also be emphasized that any analysis based on self-reports of behaviors must be cautiously interpreted, due to the well-documented trend of social desirability bias in respondent self-reports, particularly in food and weight-related research [39]. Nevertheless this study puts forth a preliminary market segmentation based on channel and message strategies that may help move Americans toward more healthy weights and lifestyles.

## Conclusion

The results of this study point to segments of the US population that may be responsive to social marketing messages that may help move them toward more healthy weights and behavior patterns. The study is among the first to apply market segmentation techniques to the domain of social marketing for obesity reduction and prevention: as such, the analysis raises some important questions for future research and gives insights into areas for public policy. Market segmentation may allow social marketing campaigns to reach specific audiences with the most effective message through the most effective media. Segmentation analysis facilitates the process of sending consumers the relevant messages specific to their lifestyles and their needs. The food industry has already employed this technique with well-documented success. Social marketing can play an important role in combating obesity and sedentary lifestyles by adopting similar techniques based upon clusters such as those identified through this research.

## Competing interests

The authors declare that they have no competing interests.

## Authors' contributions

JK conducted the analysis and drafted the original manuscript. She was the P.I. on the project grant. TR edited the manuscript, contributed to the literature review and prepared it for publication.
